# Behavior of *Pseudomonas aeruginosa* and *Enterobacter aerogenes* in Water from Filter Jugs

**DOI:** 10.3390/ijerph17218263

**Published:** 2020-11-09

**Authors:** Rossella Briancesco, Stefania Paduano, Maurizio Semproni, Luca Vitanza, Lucia Bonadonna

**Affiliations:** 1 Department of Environment and Health, Italian National Institute of Health, 00161 Rome, Italy; rossella.briancesco@iss.it (R.B.); maurizio.semproni@iss.it (M.S.); 2 Department of Biomedical, Metabolic and Neural Sciences, Section of Public Health, University of Modena and Reggio Emilia, 41125 Modena, Italy; stefania.paduano@unimore.it; 3 Department of Public Health and Infectious Diseases, Microbiology Section, “Sapienza” University of Rome, 00161 Rome, Italy; luca.vitanza@uniroma1.it

**Keywords:** drinking water, *Enterobacter*, filter jugs, *Pseudomonas*, total microbial count

## Abstract

Careless use conditions of filter jugs were applied to simulate and evaluate the behavior of two ubiquitous aquatic bacterial species, *Pseudomonas aeruginosa* and *Enterobacter aerogenes*. According to a reference protocol, nine different jugs of popular brands sold in the Italian market were used for the test. Separately, a suspension of the two bacteria was spiked in water used for filling the jugs. The concentration of the test organisms and total aerobic microbial count (TAMC) was measured daily in the filtered water along a period corresponding to the cartridge lifetime. Results showed a different trend of bacterial behavior. *E. aerogenes* was detectable exclusively on the first day after jug filling, while *P. aeruginosa* confirmed its persistence over time in all the jugs and its ability to potentially colonize surfaces and cartridges. The TAMC was detected at a concentration range from 10^2^ to 10^7^ CFU/100 mL in all the tests, high values that were not far from those raised in bottled flat natural mineral water weeks after bottling.

## 1. Introduction

In recent years, there has been an increasing demand for various household water treatment devices in European markets. Filter jugs have become alternatives to natural mineral water [1]. Water filter jugs are household water treatment devices not connected to the drinking water (DW) supply system. They are meant to be used exclusively with DW and therefore are not designed to make non-potable water potable. The improvement of the organoleptic properties of water (chlorine taste in particular) and the removal of limescale, hardness and certain metals such as lead are some of the claims made by manufacturers regarding water filter jugs [2]. Jugs employ spare cartridges that typically contain a granular mixture of ion-exchange resin and activated carbon. The resin determines the partial softening of water, while the activated carbon removes chlorine and some organic compounds. In some cases, cartridges have a silver-based compound with bactericidal properties, tending to lose their filtering capacity according to the volume of treated water and contact time with water. The cartridge lifetime is given by the manufacturers, generally corresponding to one month [3].

Some consumers show scarce confidence in tap water quality, often due to a perception of unpleasant tastes (e.g., residual chlorine). For this reason, they are able to consume natural mineral water or filtered water if they prefer [4], despite the well-consolidated European Directives [5,6] defining the criteria, parameters and values for evaluating water characteristics, guaranteeing appropriate hygienic quality. In Italy, guidelines for household water treatments are published in accordance with the Italian Decree of the Ministry of Health 25/2012 [7].

The World Health Organization has stated that “water entering the distribution system must be microbially safe and ideally should also be biologically stable” [8]. The “biological stability” requirement is scarcely defined in terms of what it actually means and how it can be achieved. Intuitively, biological stability would mean no changes occurring in the concentrations and composition of the microbial community in the water during distribution [9]. The production and distribution of biologically stable drinking water should be a priority not only for water suppliers but also for producers and users of household equipment for the treatment of drinking water. Consumers should be aware of water changes that could affect microbiological quality according to the treatment and the conservation of water in a filter pitcher.

Drinking water treatments ensure a high-quality finished product through a combination of processes aimed at removing chemical and microbiological contaminants. Drinking water is characterized by large biodiversity and conditions favoring mainly the survival of environmental microorganisms able to form biofilms. Bacterial growth is generally positively influenced by higher water temperatures, lower chlorine residuals and oligotrophy [10]. A core bacterial community can be easily observed in water and biofilms along a drinking water distribution system, independently of the water supply characteristics. Gram-negative aerobic bacteria belonging to genera *Pseudomonas*, *Acinetobacter* and other related genera usually prevail. Members of the genera *Klebsiella, Enterobacter* and *Citrobacter*, belonging to the coliform group, can be isolated at variable concentrations and successfully colonize water systems [11].

*Pseudomonas aeruginosa* is an opportunistic environmental pathogen characterized by a high degree of adaptability, capable of growing in oligotrophic waters and surviving in disinfected water. It is easily detectable in water stagnation conditions [12] and can colonize water storage tanks, faucet aerators and household devices for the treatment of drinking water [11]. Moreover, this bacterium is involved during the early stages of biofilm formation in plumbing systems due to the production of lipopolysaccharide and extracellular glycoproteins that can adhere to surfaces and facilitate the aggregation of microorganisms present in flowing water [13].

A large number of microbial cells is generally counted on surfaces in contact with drinking water [14]. Coliforms do not typically adhere first to water pipe surfaces; however, in the presence of an existing biofilm, they are able to remain attached and multiply [15].

Coliforms are used as microbial indicators for evaluating water quality and the efficiency of water treatments [16,17]. Belonging to the genus *Enterobacter*, coliforms have a strong potential for growth in aquatic environments, and drinking water can host the bacterium.

Several studies on the release of silver from pitcher filter cartridges have recently been performed [18,19], though specific controls and studies related to microbiological populations in filtered water are limited. No alerts involving water jugs have been reported via the Rapid Alert System for Food and Feed (RASFF) [20] and the Rapid Alert System for dangerous nonfood products (RAPEX) [21].

In this context, on a specific request of the Italian National Board of Health, an experimental protocol was implemented with the aim of evaluating the behavior of two ubiquitous bacterial species able to colonize surfaces: *P. aeruginosa* and *Enterobacter aerogenes*. The performance of the total aerobic microbial count (TAMC) at room temperature (around 20 °C) was taken into account.

## 2. Materials and Methods

A water filter jug comprises a container (jug), funnel, cartridge with an active filtering medium, lid and, often, a cartridge exchange indicator.

Tests were carried out on nine filter jugs of popular brands present in the Italian retail market. Water filter jug containers are made of styrene–acrylonitrile copolymers. Filtering cartridges comprise a polypropylene (PP) housing, a polyethylene terephthalate (PET) screen located inside the cartridge intended to prevent the release of filter medium particles and a filter medium comprising ion-exchange resin(s) and activated carbon. The entire filter medium or only the activated carbon is usually treated with silver. All the filter cartridges tested had a similar structure and composition with a mixture of granular silver-modified activated carbon and weak-base ion-exchange resins as the filtration matrix. The filter capacity of the water jugs ranged from 1.2 to 1.6 L, while their nominal capacity ranged from 120 to 150 L.

### 2.1. Contact Test Protocol

The validated test protocol was performed to simulate incorrect conditions of the use of filter jugs, providing the “worst case” in their management. The test period for each jug was 5 weeks in order to use 125% of the claimed cartridge capacity corresponding to the maximum treatable water volume. The test filters were set-up according to the manufacturers’ instructions for use.

After the first week of conditioning with drinking water, each pitcher was subjected to (intradaily and interdaily) contact cycles with tap water, except for the first day of each week, when they were filled with artificially contaminated water (spiked water). The spiked water consisted of tap water with sodium thiosulfate (1 mL/L of a solution at 10% *w/v*) added with one of the tested microorganisms. The filter pitchers were separately tested for the colonization ability of each microorganism. Contemporaneously, the spiked water was used as a positive control of bacteria vitality, and its bacterial concentrations were monitored in parallel with the jug-filtered water for the whole testing period. Positive controls were stored in the same conditions as water from the filter jugs.

Each intradaily contact cycle consisted of 4 consecutive contact tests, each including water filling, stagnation (30 min) and emptying. All water derived from the 4 intradaily emptying steps was collected in a single sterile container (intradaily composite sample) and mixed. During the interdaily contact cycle, jugs were kept in darkness at room temperature and the filtering cartridge remained in contact with water until the next day when the water derived from its emptying (interdaily water sample) was analyzed. This series of cycles was repeated for the first 5 days of each week followed by a stagnation period of 48 h (interweekly break). During stagnation, the tested pitcher remained in contact with the last filling water of the fifth day and the jug was kept in darkness at room temperature until the first day of the following week when the jug was emptied and the water was analyzed (interweek water sample). The whole process was replicated for 4 weeks for each jug.

### 2.2. Sampling and Analysis

For each jug, water was sampled and analyzed as follows:

- Five days a week for 4 weeks, 300 mL were collected from the intradaily composite sample after mixing for the analysis of *E. aerogenes* or *P. aeruginosa* (100 mL in triplicate); 3 mL was also collected and analyzed for TAMC (1 mL in triplicate);
- Four days a week for 4 weeks, 300 mL were collected from the interdaily water sample after mixing for the analysis of *E. aerogenes* or *P. aeruginosa* (100 mL in triplicate); 3 mL was also collected and analyzed for TAMC (1 mL in triplicate);
- Once a week for 4 weeks, 300 mL were collected from the interweekly water sample after mixing for the analysis of *E. aerogenes* or *P. aeruginosa* (100 mL in triplicate); 3 mL was also collected and analyzed for TAMC (1 mL in triplicate);
- Five days a week for 4 weeks, positive controls were tested for *E. aerogenes* or *P. aeruginosa* (1 mL in triplicate).

### 2.3. Tested Microorganisms

*E. aerogenes* (ATCC 13048, Biogenetics Diagnostics, Padua, Italy) and *P. aeruginosa* (ATCC 9027, Biogenetics Diagnostics, Padua, Italy) were the bacteria used for contact tests. They were added to spike water at a concentration ranging from 20 to 100 CFU/100 mL. The bacteria were added to jug filters one by one.

### 2.4. Microbiological Methods

*E. aerogenes* and *P. aeruginosa* were detected and enumerated in jug water samples and positive controls by standard miniaturized most probable number (MPN) methods (ISO 9308-2:2012 or Colilert 18/Quanti-Tray IDEXX test and ISO 16266-2:2018 or Pseudalert/Quanti-Tray IDEXX test, respectively) [22,23]. Analyzed samples were incubated at 36 ± 1 °C for 18 and 24 h, respectively. Results were expressed in MPN/100 mL. Both tests, designed on statistical criteria, are based on a bacterial enzyme detection technology that signals the presence of target bacteria through the hydrolysis of chromogenic or fluorogenic substrates incorporated into culture media.

TAMC was also simultaneously investigated. Water samples were analyzed by a pour plating technique on Water Plate Count (WPC, Thermo Fisher Scientific, Milano, Italy) according to ISO 6222:1999 [24]. WPC plates were incubated at 25 °C for 7 days. Results were expressed in CFU/mL.

Analysis of TAMC at 25 °C was also performed on tap water used for filling filter jugs.

### 2.5. Data Analysis

At each time, the analysis was performed in triplicate and the obtained values were averaged. The standard deviation (DS) was also calculated. Data were elaborated by analysis of variance (ANOVA) with the Bonferroni test. All statistical analyses were performed with software package IBM SPSS statistics, version 22 (IBM Corporation, Armonk, NY, USA).

## 3. Results

### 3.1. Enterobacter Aerogenes

**Figure 1:**
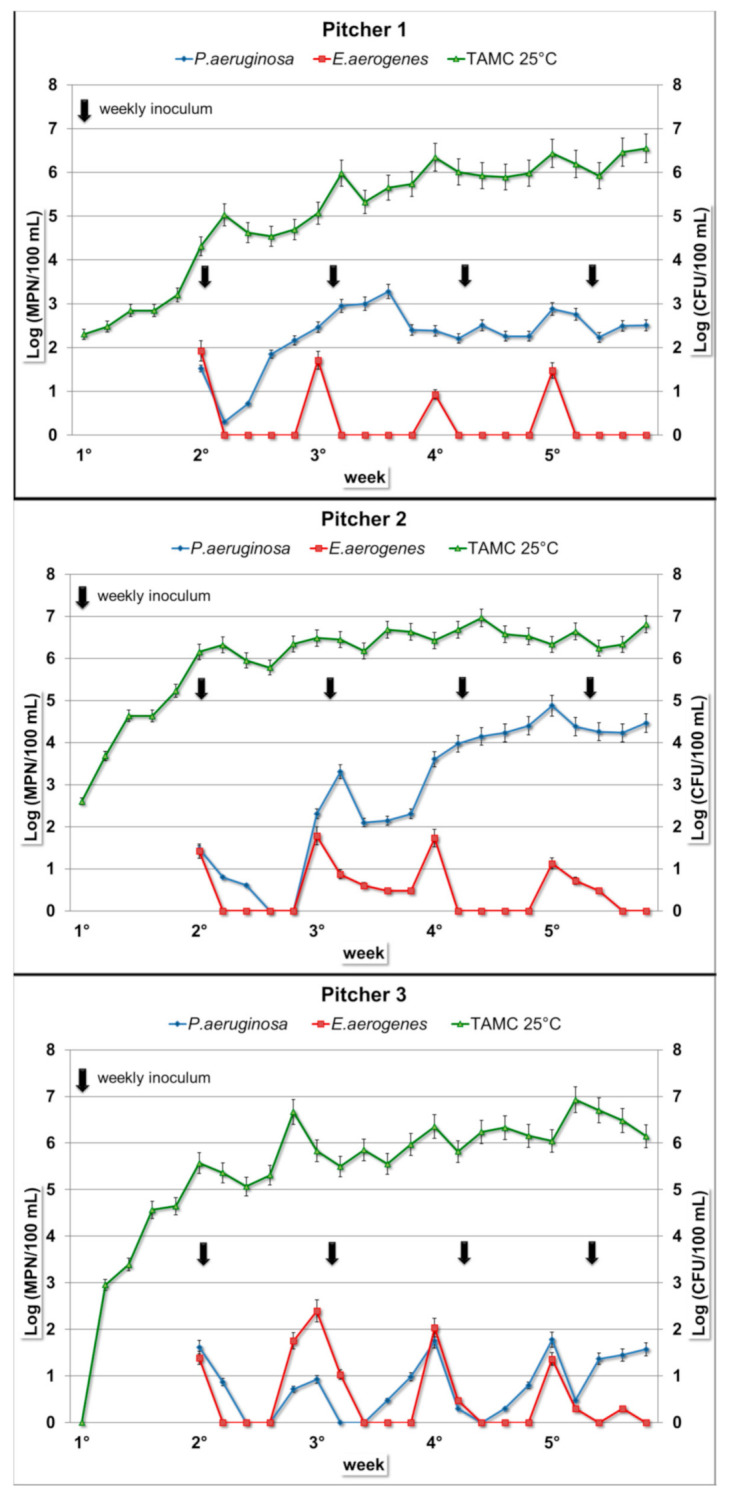
Bacterial concentration trends in daily composite samples in Filter Jugs 1–3. Arrows represent weekly inoculum for both bacteria (*E. aerogenes* and *P. aeruginosa*). The *y*-axes show: *E. aerogenes* and *P. aeruginosa* concentrations expressed in most probable number (MPN)/100 mL (left); total aerobic microbial count (TAMC) at 25 °C expressed in CFU/100 mL (right).

**Figure 2:**
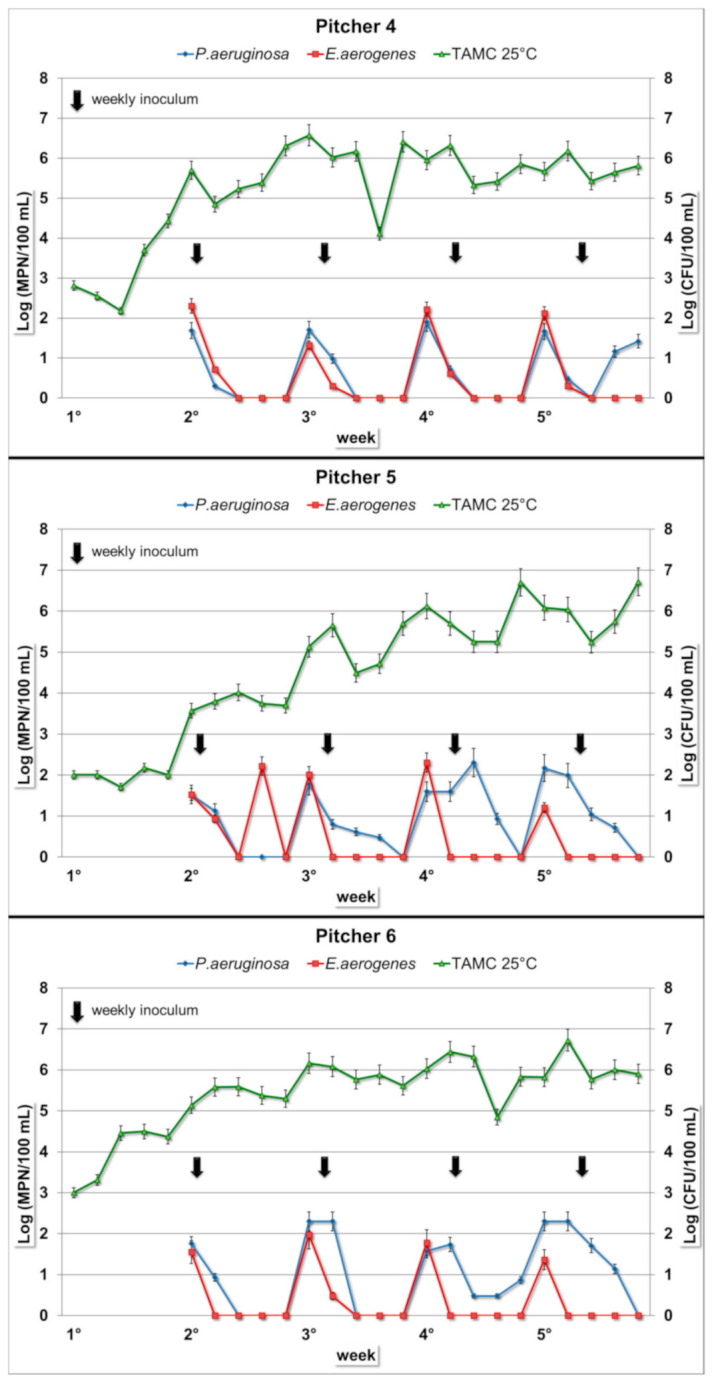
Bacterial concentration trends in daily composite samples in Filter Jugs 4–6. Arrows represent weekly inoculum for both bacteria (*E. aerogenes* and *P. aeruginosa*). The *y*-axes show: *E. aerogenes* and *P. aeruginosa* concentrations expressed in MPN/100 mL (left); TAMC at 25 °C expressed in CFU/100 mL (right).

**Figure 3:**
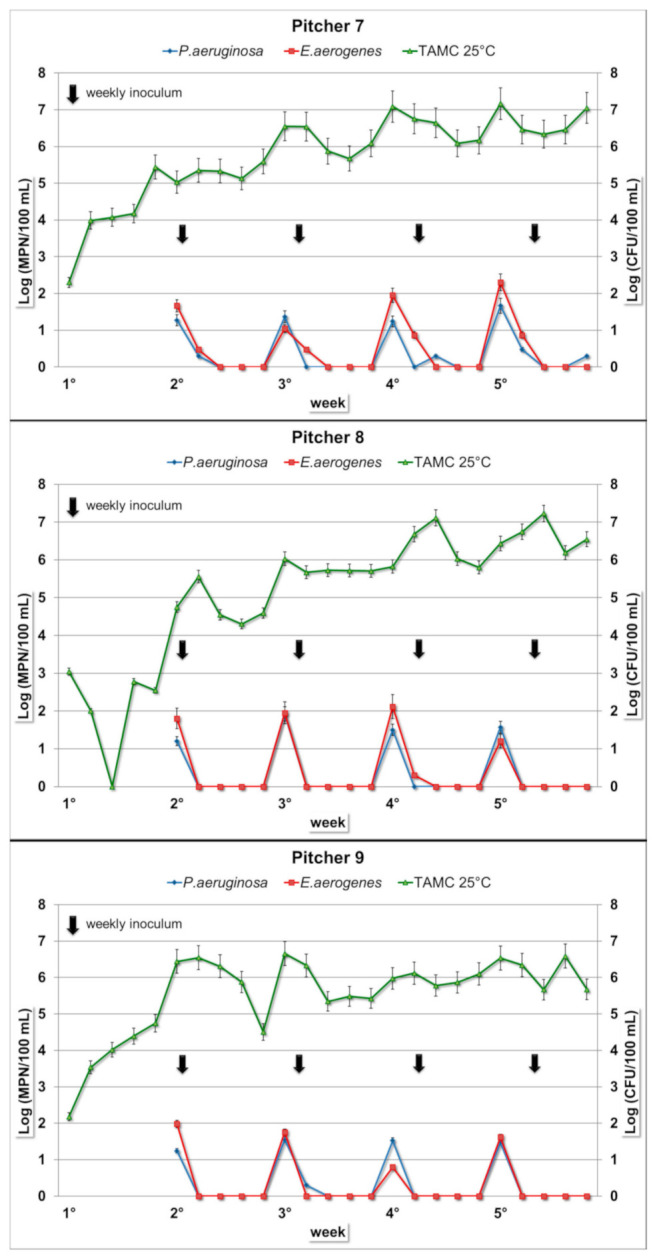
Bacterial concentration trends in daily composite samples in Filter Jugs 7–9. Arrows represent weekly inoculum for both bacteria (*E. aerogenes* and *P. aeruginosa*). The *y*-axes show: *E. aerogenes* and *P. aeruginosa* concentrations expressed in MPN/100 mL (left); TAMC at 25 °C expressed in CFU/100 mL (right).

*E. aerogenes* concentrations in the intradaily composite samples have similar values in all nine examined jugs, ranging from 0 to 10^2^ MPN/100 mL, without any statistically significant differences. The microbial load was detectable exclusively on the first day of each week. In the subsequent days, the concentration values generally remained below the limit of detection. This trend was repeated and observed in all the nine tested pitchers (Figure 1, Figure 2 and Figure 3). Similarly, in the water samples of the interdaily pause, the bacterial concentrations were detectable exclusively after the first break of each week. In the subsequent days, the results showed similar values compared to the intradaily composite samples, generally with lower values or below the limit of detection (Table 1).

After the four interweekly breaks, there were no positive samples for all the pitchers, with the only exception of one water sample collected from Pitcher 3 (*E. aerogenes*, 8.6 MPN/100 mL) (Table 1).

Moreover, the concentration of *E. aerogenes* in the spiked control water showed a low increase (2.7–5.3 times compared to the initial counts) over the first 3–4 weeks, with a 53–91% bacterial load reduction at the end of the whole testing period.

### 3.2. Pseudomonas aeruginosa

*P. aeruginosa* concentration in the intradaily composite samples showed different trends in the nine filter jugs (Figure 1, Figure 2 and Figure 3). The ANOVA test showed a global statistically significant difference among the nine filter jugs (*p* < 0.001). *P. aeruginosa* concentrations resulted significantly higher in filter jugs 1 and 2 followed by the others jugs (*p* < 0.001, Pitcher 1 vs. Pitchers 3–9; *p* < 0.001, Pitcher 2 vs. Pitchers 3–9), while no significant differences in *P. aeruginosa* concentration were detected between Pitchers 1 and 2). The composite samples of Pitcher 1 showed a progressive increase in concentration starting from the first bacterial inoculum until half of the third week, with a maximum value of 1.9 × 10^3^ MPN/100 mL. More stable values were observed over the following weeks (Figure 1). Water from Pitcher 2 showed the highest bacterial load (maximum of 7.5 × 10^4^ MPN/100 mL). In this pitcher, the bacterium concentration tended to increase over the first two weeks, remaining at a constant concentration in the last two weeks (Figure 1). Pitchers 3 and 4 displayed a similar trend with a negligible concentration or no presence of bacterium until the third and fourth weeks, respectively (Figure 1 and Figure 2). *P. aeruginosa* was detected only on the first day of each week when the pitcher was filled with the spiked water, and a slight increase was detected only in the last week. In Pitchers 5 and 6, low concentrations, mainly below the limit of detection, were maintained for the first two weeks, except for the first day of each week. In the last two weeks, concentrations of both of the pitchers showed a small increase (Figure 2). In the remaining pitchers (Pitchers 7–9), daily composites showed a detectable bacterial load exclusively on the first day of each week due to the filling with spiked water. This trend remained constant over the entire test period (Figure 3).

Water from Pitchers 1 and 2 after the interdaily break (Table 2) had higher bacterium loads compared to the composite samples (maximum of 1.6 × 10^4^ MPN/100 mL and 2.2 × 10^6^ MPN/100 mL, respectively). After the interdaily break, water from Pitchers 3 and 4 showed a trend similar to the respective composites during the first three weeks. In the last week, an increase was shown, with a maximum value of 2 × 10^2^ MPN/100 mL for Pitcher 3 and 3.3 × 10^2^ MPN/100 mL for Pitcher 4. In the case of Pitchers 5 and 6, the results of the interdaily samples showed a trend similar to their intradaily composites, with maximum values of 1.4 × 10^2^ and 2 × 10^2^ MPN/100 mL, respectively. Finally, for Pitchers 7–9 after the interdaily pause, the concentrations were low and detectable only after the break of the first day of the week (Table 2).

After the interweekly break, water samples collected from Pitchers 1 and 2 showed a trend similar to the interdaily samples with generally higher values. Water from Pitchers 3–6 had no positive results until the first two weekly breaks, while low concentrations were observed in the next two interweekly breaks. Water samples from Pitchers 7–9 showed no positive changes over all the four interweekly breaks (Table 2). In the *P. aeruginosa*-spiked water control, the bacterial concentrations showed an almost constant growth rate over the whole testing period, with a concentration rise of 5–6 orders of magnitude and high bacterial load until the end of the test (mean value: 10^6^ MPN/100 mL).

### 3.3. Total Aerobic Microbial Count at Room Temperature

For the TAMC, water samples from the nine pitchers showed the same growth trend over the entire period (Figure 1, Figure 2 and Figure 3): bacterial concentrations had a gradual increase during the five weeks, reaching concentrations of 10^6^–10^7^ CFU/100 mL. As observable in Figure 1, Figure 2 and Figure 3, the concentrations stabilized over time.

## 4. Discussion

Few studies have been conducted on the bacteriological characteristics of filtered water [25,26]. All the studies were carried out in strict compliance with the conditions of use recommended by the supplier (water periodically renewed three times a week and filtered water stored at 4 °C). The findings showed the maintenance of the microbiological quality of filtered water when there was refrigeration during the residence of water in jugs. This means that refrigeration of the jug containing the filtered water can help significantly delay the development of bacterial flora in water over time. Conversely, when jugs containing the filtered water were kept at room temperature and water was not renewed, the highest growth rates for culturable aerobic bacteria could be found. A study showed no detection of the standard indicators of fecal contamination (*E. coli* and enterococci) in filtered water [2].

Our study investigated two bacteria, one recognized as a good biofilm colonizer and the other less likely to form biofilms. The study was deliberately carried out by keeping the selected jugs under inappropriate conditions and simulating the worst-case scenario.

The data showed different trends of behavior for the two selected bacteria. In the nine pitchers, *E. aerogenes* proved its low ability to persist. Its concentrations decreased immediately after the inoculation day to values below the detection limit. This trend could be attributed to the inability of *E. aerogenes* to adhere to surfaces and persist in biofilms, although many genera of coliform bacteria are able to colonize granular-activated carbon filters [15,27].

*P. aeruginosa* is recognized as a good colonizer, able to survive in water and biofilms in poor environmental conditions (e.g., oligotrophy, different range of pH, high and low temperatures). Based on the *P. aeruginosa* concentration trend, the pitchers were divided into three different groups. In the first group (Pitchers 1 and 2), the microorganism was present in the filtered water, likely colonizing filters and internal surfaces, from the first week, increasing its concentration in water throughout the study period. In the second group (Pitchers 3–6), *P. aeruginosa* was found within the filtered water of Pitchers 3–6 only at the end of the study period, probably due to slower colonization of cartridges and surface. In the remaining pitchers (Pitchers 7–9), the water did not contain *Pseudomonas* other than after each filling.

Interestingly, all the selected jugs had filter cartridges with a silver-based compound. However, the filtered water had different quality characteristics with regard to *Pseudomonas*. It is conceivable that some jugs had a greater tendency to favor the *Pseudomonas* colonization of filters and surfaces.

The antimicrobial activity of the silver ion is widely used to control bacterial growth in a variety of medical applications and drinking water for disinfection purposes [28]. The efficacy of the water disinfection process depends mainly on Ag ion concentrations, contact time and specific microbial characteristics. In a study on the efficacy of silver ions (up to 100 μg/L) against *P. aeruginosa* in synthetic drinking water, the authors found a 4-log reduction of *P. aeruginosa* after a three-hour contact time [29]. A 5-log reduction in *P. aeruginosa* was similarly observed after a 12 h contact time, with 80 μg/L Ag, against 3 × 10^6^ CFU/mL of *P. aeruginosa*. Based on these data, it is presumable that the *c* × *t* value available in the colonized cartridges’ effluents was not high enough to ensure an efficacy action by Ag ions. The exclusive conformation, as well as the porosity of the filters, could have differently supported the biofilm formation and consequently the growth of *P. aeruginosa*. A reduced efficacy of Ag ions on biofilm bacteria has been reported by Silvestry-Rodriguez et al., who found that silver (100 μg/L) was ineffective in reducing biofilm formation in drinking water distribution systems [30]. Data obtained from chemical analyses carried out in parallel with our microbiological analyses on the same jugs and water showed variable concentrations of silver with values ranging from 17 to 39 µg/L (≤1 µg/L in the initial water) [31]. The data confirm the ability of *P. aeruginosa* to adapt to oligotrophic environmental conditions, typical of many drinking water systems, and to persist and multiply in biofilm over a long period [32,33].

Studies performed on healthy human volunteers observed that colonization by *P. aeruginosa* (not the disease) of subjects required an oral dose of more than 1.5 million bacteria, demonstrating that *P. aeruginosa* generally does not infect healthy organisms, while immunocompromised persons are certainly more at risk [34,35]. The ingestion of water containing *P. aeruginosa* can be considered a risk for deeply immunocompromised individuals and does not seem to be a risk for the general population. The infectious dose for healthy individuals has been calculated to be in a range between 10^6^ and 10^10^ CFU/mL, which, considering the pathogenic opportunistic nature of the bacterium, may become much lower in the case of immunocompromised individuals [36]. Therefore, the ascertained ability of *P. aeruginosa* to colonize some of the jug filters tested makes the use of water jugs a condition of risk for vulnerable population groups considering the lower infectious dose for immunosuppressed individuals.

In filtered water from all the pitchers, the TAMC was detected at concentrations ranging from 10^2^ to 10^7^ CFU/100 mL. Water stagnation in all the jugs caused a general deterioration of the microbiological quality with a gradual but constant increase over time. The TAMC, as a microbiological parameter, has little value as an indicator of pathogen presence [11]. In fact, most of the microorganisms growing under the term TAMC generally include members of the natural (typically nonhazardous) microbiota of water and biofilm and, in some instances, organisms from diverse pollutant sources. In distribution systems, increasing numbers of TAMC can indicate a deterioration in cleanliness, possible stagnation phenomena and the potential development of biofilms. Under these experimental conditions, the TAMC concentration is comparable to those of water maintained in static conditions in containers or bottles [37]. The high concentrations reached in this study can be explained by an increase of vital bacteria into the biofilm with periodic detachment. It has been demonstrated that plastic materials can be colonized in higher densities compared to the total bacterial cell count grown on steel and copper in bank-filtered water [38]. The presence of activated carbon inside jug filters may contribute to the fast and increased colonization of bacteria. The biofilm on the granular carbon particles is described as a monolayer facilitating an intensive contact between microorganisms and substrates dissolved in water or fixed in carbon particle pores. The granular-activated carbon, used in the household treatment devices, permits the nutrients accumulation and neutralizes residues of disinfectants, providing an ideal environment for microbial growth and consequent worsening of the microbiological quality of water [11,39].

Interestingly, a study was conducted in order to examine the possible adverse effects of jug filters on water quality [26]. This study concluded that there was no evidence of microbiologically adverse effects related to the use of filter pitchers, even in test conditions that simulate possible contamination with pathogenic microorganisms (*E. coli* and *Salmonella*). In particular, *Salmonella* and *E. coli* deliberately spiked into the feed water did not survive in the filtered water or colonize the filter media. It was found that these organisms were only present, if at all, in the first filtrate following inoculation. The TAMC levels tended to be higher in filtered water compared to the feed, particularly with high-nutrient test water. There was no indication of an “explosion” in TAMC levels. Overall, this study concluded that there was no evidence that jug water filter systems have adverse effects on microbial water quality, even when tested under conditions simulating abuse and contamination. Similarly, our results do not indicate a real health risk in these experimental conditions; however, an increase of microbial concentration was observed for *P. aeruginosa* and TAMC.

## 5. Conclusions

Our results showed a different trend of bacterial behavior. In fact, the concentrations of *P. aeruginosa* and *E. aerogenes* varied according to the specific characteristics of each type of bacterium, more or less inclined to persist on surfaces and filter cartridges. The obtained data are encouraging because, despite simulated inappropriate management of the jugs, no serious health risk emerged, although an increase of concentrations was calculated for *P. aeruginosa* and TAMC. On the one hand, the TAMC showed a regular increase with a rise in the concentration of 3–4 order of magnitude during the five experimental weeks and, on the other hand, a concentration increase of *P. aeruginosa*, a recognized opportunistic pathogen.

Finally, further studies would be useful for investigating the possible formation of biofilm in the cartridges, and to check the effects on the filtering cartridges and filtered water in the presence of contemporary microbial contamination.

## Figures and Tables

**Table 1:** *E. aerogenes* concentrations from interdaily and interweekly stagnation water (9 pitchers tested for 4 weeks).

*E. aerogenes* Pitcher (n.)	Interdaily Stagnation (16 Per Pitcher) *	Interweekly Stagnation (4 Per Pitcher) **
	Mean ± SD (min–max) MPN/100 mL	Mean ± SD (min–max) MPN/100 mL
1	8.9 ± 2.1 × 10^1^	0
	(0.0–7.8 × 10^1^)	(0.0–0.0)
2	6.0 ± 1.2 × 10^1^	0
	(0.0–4.3 × 10^1^)	(0.0–0.0)
3	4.4 ± 7.9	2.2 ± 4.3
	(0.0–2.9 × 10^1^)	(0.0–8.6)
4	4.6 × 10^1^ ± 8.1 × 10^1^	0
	(0.0–2.0 × 10^2^)	(0.0–0.0)
5	1.8 × 10^1^ ± 3.9 × 10^1^	0
	(0.0–1.3 × 10^2^)	(0.0–0.0)
6	1.2 × 10^1^ ± 3.1 × 10^1^	0
	(0.0–1.0 × 10^2^)	(0.0–0.0)
7	3.3 × 10^1^ ± 6.3 × 10^1^	0
	0.0–2.0 × 10^2^	(0.0–0.0)
8	1.6 × 10^1^ ± 3.4 × 10^1^	0
	0.0–1.0 × 10^2^	(0.0–0.0)
9	7.1 ± 1.5 × 10^1^	0
	0.0–4.3 × 10^1^	(0.0–0.0)

* interdaily breaks are 16/pitcher and analyses were performed in triplicate (*N* = 48). ** interweekly breaks are 4/pitcher and analyses were performed in triplicate (*N* = 12). SD: standard deviation.

**Table 2:** *P. aeruginosa* concentrations from interdaily and interweekly stagnation water (9 pitchers tested for 4 weeks).

P. aeruginosa Pitcher (n.)	Interdaily Stagnation (16 Per Pitcher) *	Interweekly Stagnation (4 Per Pitcher) **
	Mean ± SD (min–max) MPN/100 mL	Mean ± SD (min–max) MPN/100 mL
1	4.4 × 10^3^ ± 4.4 × 10^3^	5.9 × 10^3^ ± 3.5 × 10^3^
	(6.4–1.6 × 10^4^)	(0.0–9.1 × 10^3^)
2	5.3 × 10^5^ ± 7.0 × 10^5^	2.1 × 10^6^ ± 2.6 × 10^6^
	(0.0–2.2 × 10^6^)	(0.0–5.6 × 10^6^)
3	2.6 × 10^1^ ± 4.9 × 10^1^	1.8 ± 2.5
	(0.0–2.0 × 10^2^)	(0.0–5.3)
4	5.5 × 10^1^ ± 9.5 × 10^1^	5.0 × 10^2^ ± 1.0 × 10^3^
	(0.0–3.3 × 10^2^)	(0.0–2.0 × 10^3^)
5	3.2 × 10^1^ ± 5.3 × 10^1^	4.2 ± 8.4
	(0.0–1.4 × 10^2^)	(0.0–1.7 × 10^1^)
6	5.6 × 10^1^ ± 7.6 × 10^1^	8.4 ± 1.3 × 10^1^
	(0.0–2.0 × 10^2^)	(0.0–2.7 × 10^1^)
7	8.9 ± 1.7 × 10^1^	0
	0.0–5.3 × 10^1^	(0.0–0.0)
8	1.0 × 10^1^ ± 2.6 × 10^1^	0
	0.0–1.0 × 10^2^	(0.0–0.0)
9	6.7 ± 1.2 × 10^1^	0
	0.0–3.2 × 10^1^	(0.0–0.0)

* interdaily breaks are 16/pitcher and analyses were performed in triplicate (*N* = 48). ** interweekly breaks are 4/pitcher and analyses were performed in triplicate (*N* = 12). SD: standard deviation.
